# Nocebo Effect During Letrozole Desensitization: A Case Report

**DOI:** 10.7759/cureus.87841

**Published:** 2025-07-13

**Authors:** Asimina Koulouridi, Panayiota Nikiphorou, Panayiota Gelastou, Constantinos Pitsios

**Affiliations:** 1 Department of Oncology, German Oncology Center, Limassol, CYP; 2 Internal Medicine Clinic, Nicosia General Hospital, State Health Services Organization, Nicosia, CYP; 3 Department of Allergy and Immunology, Medical School, University of Cyprus, Nicosia, CYP; 4 Allergy Outpatient Clinic, Nicosia General Hospital, State Health Services Organization, Nicosia, CYP

**Keywords:** aromatase inhibitor therapy, breast cancer management, drug allergy documentation, drug desensitization, drug provocation test, drug-related side effects and adverse reactions, facticious disorder, healthcare ethics, letrozole

## Abstract

This report describes the case of a 46-year-old woman with breast cancer and a history of multiple drug hypersensitivity reactions, who developed atypical allergic-like symptoms with three different adjuvant endocrine therapy regimens. As the use of an aromatase inhibitor was considered essential for her treatment, a three-day desensitization protocol to letrozole was implemented. During the protocol, she developed pruritic erythema following placebo administration. Despite this, the protocol was completed, with only mild side effects (oropharyngeal pruritus and headache), which were managed symptomatically. A diagnosis of nocebo reaction was disclosed to the patient after the desensitization process. She continued daily treatment with 2.5 mg of letrozole without further hypersensitivity complications.

## Introduction

Breast cancer remains the most common cancer among women worldwide, accounting for approximately 30% of all female cancers [1]. The majority of breast cancers are estrogen receptor-positive, and adjuvant endocrine therapy (ET) is a key therapeutic approach for these patients. Options for adjuvant ET include tamoxifen or an aromatase inhibitor, which may be steroidal (exemestane) or non-steroidal (letrozole or anastrozole) [2].

Nearly all breast cancer patients receiving ET experience side effects, with vasomotor symptoms (such as hot flashes and night sweats) and musculoskeletal complaints (joint pain, stiffness, arthralgia) being the most frequent [3]. Although allergic reactions to ET medications are unusual, there are some reports of hypersensitivity reactions to anastrozole and letrozole, and desensitization protocols have been developed to safely continue treatment [4-7].

We hereby report the case of a patient who was hospitalized to undergo desensitization to letrozole. An unusual nocebo reaction was observed during the desensitization, highlighting that underlying psychological mechanisms can sometimes contribute to reported side effects of antitumoral drugs.

## Case presentation

A 46-year-old female was diagnosed with locally advanced breast cancer, characterized by TNM classification pT2pN1(1/16), grade 2 (GR2), ER+, PR+, HER2-, Ki 67 10%, and classified as luminal A, with a low-risk MammaPrint profile. Based on this diagnosis, and although she was over 35 years old, it was decided to follow a treatment plan appropriate for premenopausal, high-risk disease. She underwent a wide local excision with left axillary lymph node dissection, followed by radiotherapy. Subsequently, she was placed on ET with tamoxifen, combined with ovarian function suppression using goserelin.

Her medical history included cardiac arrhythmias treated with cardiac ablation, and she is currently being monitored for idiopathic thrombocythemia, which is managed with aspirin. The patient reported food and respiratory allergies, as well as unconfirmed drug hypersensitivity reactions to ipratropium inhaler and vitamin D.

After starting tamoxifen, she experienced an acute reaction 20 minutes after the first dose, presenting with generalized maculopapular pruritic exanthema, which was not relieved by antihistamines. Despite this, treatment was continued. On the third day, the patient developed edema of the extremities with worsening pruritus along with throat tightness, leading to tamoxifen discontinuation.

Exemestane was initiated two weeks later, but pruritus developed on the first day. On the third day, she developed a burning sensation on the face, pronounced pruritus, a feeling of near-fainting, and tremor. The patient presented to the emergency room, where an initial evaluation raised a clinical suspicion of a panic attack, as no objective signs or findings were observed to support a drug-related adverse reaction. Tryptase was measured to exclude a mast-cell disorder, but it was within normal levels.

Two weeks before her visit to the Allergy Department, she started letrozole. Pretreatment with levocetirizine (5mg twice daily) was initiated two days prior to letrozole intake. However, about two hours after the first dose, she developed a papular pruritic rash, which led to the discontinuation of letrozole.

Upon allergy evaluation, a detailed anamnesis revealed a history of food allergy to shrimp (anaphylaxis) and fish (pruritus), allergic rhinitis, rash following tetanus immunization, and an itchy rash after the administration of MRI contrast media. Prick-to-prick skin tests and patch tests (10% in petrolatum) to tamoxifen, exemestane, and letrozole were negative. Repeated serum tryptase and total IgE were also within normal levels.

Desensitization to letrozole was recommended, and the patient was admitted during the following week. A desensitization protocol, based on a previously published anastrozole protocol, was followed (Table 1) [6]. A bland capsule with placebo (no active substance) and capsules with different concentrations of letrozole were prepared by the pharmacology laboratory of the medical school. Ten minutes after the administration of the initial bland capsule, she developed pruritic erythema on both arms and the supraclavicular area. An ethical dilemma arose as to whether we should reveal to the patient that she had reacted to a placebo. The decision had to be made quickly, and, thus, in order to not jeopardize the successful introduction of letrozole into her treatment, it was decided to proceed without disclosing that she experienced a nocebo effect.

**Table 1:** Doses of letrozole, adverse reactions, interventions, and outcomes during the three-day desensitization protocol

Day	Dose (mcg)	Adverse reactions	Intervention	Total daily dose (mg)
1	Placebo	Pruritic erythema	None	0.775
	25	-	-	
	50	Oropharyngeal pruritus	Cetirizine	
	100	-	-	
	200	-	-	
	400	-	-	
2	400	-	-	2.5
	500	-	-	
	500	Headache	Paracetamol	
	500	-	-	
	600	-	-	
3	1,250	-	-	2.5
	1,250	-	-	

Desensitization continued with the administration of 25mcg of letrozole, and symptoms gradually subsided. She reported oropharyngeal itching after the third capsule (day 1), and cetirizine 10mg was given as symptomatic treatment. She completed the three-day protocol, experiencing only mild headache. She was discharged with instructions to continue letrozole 2.5mg daily.

Upon re-evaluation, she disclosed past traumatic experiences and persistent high stress levels. She was informed that her initial reaction was to a placebo, and she initially declined psychological evaluation and support but ultimately accepted the intervention. Six months post-desensitization, the patient demonstrated good compliance with the treatment. She reported elevated blood pressure since hospitalization, which was managed by her general practitioner.

## Discussion

Although allergic reactions to aromatase inhibitors are extremely rare, desensitization is occasionally required. Rodrigues et al. described two cases of hypersensitivity to anastrozole, where a three-day desensitization protocol was successfully implemented [6]. Another report involved desensitization to letrozole in a patient with reactions to letrozole, anastrozole, and tamoxifen. Due to side effects, the protocol was repeated after the introduction of omalizumab and was ultimately tolerated [7].

Multiple drug intolerance syndrome is relatively uncommon but is strongly associated with female gender, mental health and somatic disorders, increased psychotropic medication use, and greater healthcare utilization [8]. “Polyallergic” patients have 8.5-fold the odds of a functional-somatic-syndrome diagnosis, when compared to patients with non-allergic subjects. Our polyallergic patient’s anamnesis of multiple drug intolerance was not confirmed by allergy testing and appeared to be the result of psychosomatic manifestations. However, psychosomatic diagnosis requires the exclusion of other causes, and, thus, allergology consultation was crucial.

In patients with suspected drug hypersensitivity reactions and negative skin tests, placebo-controlled drug provocation tests (PCDPTs) are the diagnostic gold standard. It has been reported that 30-56% of patients undergoing drug provocation testing to chemotherapeutic agents have negative results [9]. On the other hand, drug desensitization remains the primary therapeutic strategy for managing chemotherapeutic hypersensitivity. Although more time-consuming than the provocation test, its aim is therapeutic rather than diagnostic.

In our case, while a PCDPT might have been considered a necessary diagnostic step, a positive test would simply have indicated that the reported reactions were not immune-mediated. The occurrence range of nocebo effect in PCDPT is 3-21%, depending on factors such as the underlying disease and the experience of previous side effects, which foster an expectation of new troublesome reactions [10,11]. In a retrospective study, 8.17% of patients reacted to placebo and 41% of them reacted again to placebo during subsequent PCDPT, with symptoms similar to their initial reaction [12]. This shows that even if we had informed our patient that her reactions were not immune-mediated and proceeded directly to the therapeutic dose, she would most likely have reacted again, undermining the regular administration of letrozole.

Following ethical guidelines, our patient had provided written informed consent before PCDPT and had also given consent for publication of her case. The ethical dilemma concerned the timing of disclosure of the placebo response. Given the goal of ensuring successful letrozole administration, we completed the protocol without immediate disclosure; the patient was later informed by her oncologist about her nocebo reaction and on the suspicion that her previous hypersensitivity reactions were also non-immunological. Psychological consultation and follow-up were recommended.

## Conclusions

This case highlights the diagnostic value of placebo in allergology and the importance of multidisciplinary care of oncologic patients. While oncology specialists may coordinate treatment, optimal care requires collaboration among various experts. Tumor boards and specialized oncology centers can facilitate such collaborative management.
